# Long-Term Follow-Up after Adoptive Transfer of BK-Virus-Specific T Cells in Hematopoietic Stem Cell Transplant Recipients

**DOI:** 10.3390/vaccines11040845

**Published:** 2023-04-14

**Authors:** Michael Koldehoff, Britta Eiz-Vesper, Britta Maecker-Kolhoff, Nina K. Steckel, Ulf Dittmer, Peter A. Horn, Monika Lindemann

**Affiliations:** 1 Zotz Klimas, MVZ Düsseldorf, 40210 Düsseldorf, Germany; koldehoff@zotzklimas.de; 2 Department of Hematology and Stem Cell Transplantation, University Hospital Essen, University Duisburg-Essen, 45147 Essen, Germany; nina.steckel@rub.de; 3 Institute of Transfusion Medicine and Transplant Engineering, Hannover Medical School, 30625 Hannover, Germany; eiz-vesper.britta@mh-hannover.de; 4 Department of Pediatric Hematology and Oncology, Hannover Medical School, 30625 Hannover, Germany; maecker.britta@mh-hannover.de; 5 Department of Medicine, University Hospital Knappschaftskrankenhaus Bochum, Ruhr University Bochum, 44892 Bochum, Germany; 6 Institute for Virology, University Hospital Essen, University Duisburg-Essen, 45147 Essen, Germany; ulf.dittmer@uk-essen.de; 7 Institute for Transfusion Medicine, University Hospital Essen, University Duisburg-Essen, 45147 Essen, Germany; peter.horn@uk-essen.de

**Keywords:** BK virus, hematopoietic stem cell transplantation, treatment with virus-specific T cells, immunosuppression, cidofovir, JC virus, CMV, EBV, monitoring of T-cell immunity, ELISpot

## Abstract

The BK virus (BKV) causes severe hemorrhagic cystitis in hematopoietic stem cell transplant (HSCT) recipients. To eliminate reactivated BKV, symptomatic patients can be treated with a reduction of the immunosuppressive therapy, with the antiviral drug cidofovir, or with virus-specific T cells (VSTs). In the current study, we compared the effect of VSTs to other treatment options, following up specific T cells using interferon-gamma ELISpot assay. We observed BKV large T-specific cellular responses in 12 out of 17 HSCT recipients with BKV-related cystitis (71%). In recipients treated with VSTs, 6 out of 7 showed specific T-cell responses, and that number in those without VSTs was 6 out of 10. In comparison, 27 out of 50 healthy controls (54%) responded. In HSCT recipients treated for BKV-related cystitis, absolute CD4+ T-cell numbers and renal function correlated with BKV-specific cellular responses (*p* = 0.03 and 0.01, respectively). In one patient, BKV-specific cellular immunity could already be detected at baseline, on day 35 after HSCT and prior to VSTs, and remained increased until day 226 after VSTs (78 vs. 7 spots increment). In conclusion, the ELISpot appears to be suitable to sensitively monitor BKV-specific cellular immunity in HSCT recipients, even early after transplantation or in the long term after VSTs.

## 1. Introduction

Protection of hematopoietic stem cell transplant (HSCT) recipients from infection remains challenging owing to severe immunosuppression after transplantation. The BK virus (BKV), a DNA virus also called Betapolyomavirus hominis, is a cause of severe hemorrhagic cystitis and of nephropathy in patients treated with allogeneic HSCT [1]. BKV was first isolated in 1971 from the urine of a kidney transplant recipient with the initials B.K. The virus can be transmitted via smear infections with urine, droplet infection, or contaminated drinking water and, in the adult population, the infestation rate with BKV is greater than 80% [2]. In HSCT patients, the gastrointestinal tract could be identified as a major persistence site, apart from the uroepithelium [3]. Fecal BKV excretion was detectable in 40% of these patients [3].

BKV remains persistent after primary infection and may reactivate during immunosuppression. In immunocompetent individuals, however, BKV infection is usually asymptomatic. Nevertheless, a study in 400 healthy blood donors showed that 7% shed the virus into the urine [4], indicating that BKV transiently escapes from immune control even in immunocompetent individuals [5]. In immunocompromised individuals, BKV replication increases in rate and magnitude, progressing to hemorrhagic cystitis and nephropathy in 5–50% of allogeneic HSCT recipients [6,7,8]. The incidence of BKV-related hemorrhagic cystitis after allogeneic HSCT is highly variable among adult (up to 50%) and pediatric recipients (up to 25%), especially in the setting of graft versus host disease (GvHD) [7,8]. Other studies reported a profound increase in the incidence of BKV-related hemorrhagic cystitis in the setting of allogeneic haploidentical HSCT and after utilizing posttransplant cyclophosphamide (PT/Cy) as GvHD prophylaxis. On the one hand, PT/Cy leads to direct damage of the bladder mucosa and, on the other hand, to delayed T-cell reconstitution with a deficiency of BKV-specific T cells in the circulation, resulting in BKV replication due to a lack of immune surveillance [9,10,11,12].

Several therapeutic approaches have been used for the treatment of BKV-related hemorrhagic cystitis. Reduction or cessation of immunosuppression has been considered an attempt to enhance anti-BKV immunity, but a favorable risk–benefit ratio in allogeneic HSCT recipients must be weighed against the risk of donor alloreactivity to the host, and the severity of GvHD must be considered. Alternatively, patients can be treated with the antiviral drug cidofovir, a cytosine derivative of an acyclic nucleoside-phosphonate analogue, which has broad-spectrum activity against many DNA viruses including BKV [13]. Of note, BKV does not have a DNA polymerase. However, treatment with cidofovir can lead to nephrotoxicity and neutropenia. Finally, virus-specific T cells (VSTs), which play a key role in the elimination of reactivated BKV infection, are a promising treatment option [14,15,16,17,18,19,20,21]. Whereas the generation and clinical impact of BKV-specific VSTs in the HSCT setting is now described by several groups [16,17,18,19,20,21,22], data on the monitoring of BKV-specific T cells in HSCT recipients are still scarce [17,19]. The majority of studies reported excellent clinical responses, with a decrease in viral load and symptomatic improvement in 74–100% of patients [16,17,19]. However, Holland et al. described one patient with severe cytokine release syndrome following BKV-specific VSTs [22]. Tzannou et al. and Olsen et al. determined BKV-specific cellular immunity either by ELISpot [17] or by intracellular cytokine assays [19] and presented follow-up data until month 3 after VSTs.

In the current study, we sequentially analyzed HSCT recipients with BKV-related cystitis, who were treated by a reduction of immunosuppressive drugs, with cidofovir and/or with VSTs. As compared with the previous studies on cellular responses in HSCT recipients after infusion of BKV-specific VSTs [17,19], follow-up was longer (up to 910 days, i.e., 30 months, after VSTs) and we show in parallel the time courses of BKV-specific T-cell immunity, viral load, and immunosuppressive medication. In contrast to the two previous studies [17,19], we monitored cellular BKV-specific immunity using IFN-γ ELISpot assays, where we compared responses to various BKV antigens. As five HSCT recipients received VSTs also directed against cytomegalovirus (CMV) and/or Epstein–Barr virus (EBV), we monitored cellular immunity against CMV and EBV in parallel.

It was our aim to compare the effect of VSTs and other treatment options especially on antiviral T-cell immunity, which was followed up over the long term. Moreover, we analyzed whether co-variates such as leukocyte subpopulations, immunoglobulins, or renal function correlated with BKV-specific cellular immunity. Finally, we assessed whether BKV-specific immunity differed between 17 HSCT recipients with dysuria and BKV-related cystitis, 5 HSCT recipients with dysuria but without BKV-related cystitis, and 50 healthy controls.

## 2. Materials and Methods

### 2.1. Volunteers

The study comprises 22 adults treated with allogeneic HSCT (median age 52 years, range 21–77), of whom 4 were female (Table 1). Two patients received their stem cell graft from a matched sibling donor, 4 from a human leukocyte antigen (HLA) haploidentical related donor, and 16 from an unrelated donor. All patients were recruited at the University Hospital Essen (Essen, Germany) when they presented with current or previous dysuria and agreed to participate in the study. Enrollment in the study—which comprised the monitoring of virus-specific cellular immunity—was offered to all patients in our outpatient clinic. In addition to measuring immunity to BKV, we also included JC virus (JCV) as a control because it can also cause cystitis or nephropathy and is structurally related to BKV [23,24]. The median interval between HSCT and BKV-related cystitis was 35 days (range 1–3359 days, i.e., 9.2 years), that between the onset of cystitis and the first ELISpot analysis was 28 days (range 0–973), and that between HSCT and study inclusion was 85 days (range 26 days–12 years). As a control group, we tested 50 age-matched, related HSCT donors (median age 49 years, range 21–66). The study was conducted according to the guidelines of the Declaration of Helsinki and approved by the Ethics Committee of the University Hospital Essen, Germany (19-9039-BO). Informed consent was obtained from all subjects involved in the study.

### 2.2. Determination of Antiviral Cellular Immunity by ELISpot

Nine milliliters of heparinized blood were collected and peripheral blood mononuclear cells (PBMC) were separated by Ficoll gradient centrifugation. The numbers of PBMC were determined by an automated hematology analyzer (XP-300, Sysmex, Norderstett, Germany). Duplicate cultures of 400,000 freshly isolated PBMCs were grown with and without three peptide mixtures of BKV (large T (LT) peptides from AID, Autoimmun Diagnostika GmbH, Strassberg, Germany; LT peptides from JPT Peptide Technologies GmbH, Berlin, Germany; peptides of viral protein 1 (VP1) from AID).

The LT peptide mix from AID contains 75 peptides and covers the complete LT antigen. It was used at a concentration of 1.3 µg/mL per 20 mer peptide. The LT peptides mix from JPT contains 170 peptides and also spans the complete antigen. Each of the 15 mer peptides, which overlap by 11 amino acids, was used at a concentration of 1 µg/mL. The VP1 antigen consists of 40 peptides and covers the complete antigen. Like the LT antigen from AID, it was used at a concentration of 1.3 µg/mL per 20 mer peptide.

For comparison, cells were stimulated with 1.3 µg/mL per 20 mer peptide of JCV (AID), which consists of six peptides. Of note, the JCV peptides, partly derived from VP1 of JCV, were selected using several tools predicting T-cell epitopes, e.g., http://tools.iedb.org/main*/* accessed on 20 February 2023, and they are considered as highly specific. Nevertheless, owing to their localization, a cross-reaction with BKV cannot be ruled out completely [25]. The production of IFN-γ was determined using pre-coated ELISpot plates and a standardized detection system (T-Track^®^ ELISpot kit, Mikrogen GmbH, Neuried, Germany). PBMCs were incubated in 150 µL AIMV medium (Gibco, Grand Island, NE, USA) at 37 °C for 1 day. Stimulation with the T-cell mitogen phytohemagglutinin (PHA, 4 µg/mL) served as a positive control. These conditions could be defined as optimal and were used if not stated otherwise. In order to optimize the ELISpot conditions, we used 250,000 and 400,000 freshly isolated PBMC per culture, titrated viral antigens (BKV LT peptide mix, 0.8–1.7 µg/mL per peptide, AID; BKV LT peptide mix, 0.1–10 µg/mL peptide, JPT), and performed the cell cultures for 1–3 days. BKV VP1 was not titrated, but used at the concentration recommended by the manufacturer, which was the same as for BKV LT (1.3 µg/mL).

In a subset of six HSCT recipients (of which five received also CMV- and/or EBV-specific VSTs), cellular responses to CMV and/or EBV were determined in parallel, with 200,000 PBMCs and an ELISpot protocol described previously [26]. We either used duplicates with two T-activated^®^ CMV proteins, immediate early antigen-1 (IE-1) and phosphoprotein 65 (pp65), according to the manufacturer’s instructions (T-Track^®^ CMV, Mikrogen, Neuried, Germany), or an EBV lysate (whole virus, R02100, Meridian Bioscience, Cincinnati, OH, USA) plus the T-Track^®^ ELISpot Basic Kit strip (Mikrogen, Neuried, Germany).

Colorimetric detection of cytokine secreting cells was performed according to the manufacturer’s instructions (Mikrogen). Spot numbers were analyzed by an ELISpot reader (Fluorospot, AID, Autoimmun Diagnostika GmbH, Strassberg, Germany). Apart from considering individual concentrations of viral antigens, we determined the spots increment, i.e., we determined median values of virus-specific responses and subtracted the median of negative (unstimulated) controls. The cut-off definition for positive responses was based on negative control values and on the consideration that threefold higher values for virus-stimulated versus unstimulated cells are frequently considered as a positive response in cellular assays. In HSCT recipients, the negative controls for IFN-γ had a median of 0.5 spots (range 0–8) and its threefold standard deviation was 3 × 1.57 spots = 4.71 spots (which we considered as the background of the negative controls). In healthy individuals, the negative controls for IFN-γ had a median of 0.5 spots (range 0–5) and its threefold standard deviation was 3 × 0.91 spots = 2.73 spots. Based on these numbers, we chose a value of at least five spots increment as the criterion for positivity. In all patients with at least a five spots increment, the virus-stimulated responses were more than threefold higher than the unstimulated controls.

### 2.3. Quantitative Real-Time PCR

Viral load for BKV was determined as described previously, using the RealStar BKV PCR kit 1.0 (Altona Diagnostics, Hamburg, Germany) and the Light Cycler 96 system (Roche, Basel, Switzerland) [27]. CMV- or EBV-DNA was quantified with the fully automated Abbott m2000rt real-time PCR system using the Abbott RealTime CMV or EBV amplification reagent kit according to the manufacturer’s instructions (Abbott, Wiesbaden, Germany). The manufacturer reported the lower limit of quantification (LLQ) as 360 IU/mL (BKV), 65 IU/mL (CMV), and 150 IU/mL (EBV).

### 2.4. Flow Cytometry

We collected whole peripheral blood samples from patients with BKV viremia and the samples were analyzed at the BMT Flow Cytometry Laboratory, University Hospital Essen. PBMCs were isolated using an automatic red blood cell lysing system (TQ-Prep, Beckman Coulter, Brea, CA, USA), washed with fluorescence-activated cell sorting buffer, and stained for surface markers. No intracellular staining was performed. Flow cytometric analysis of the patient’s immune status was performed on a NAVIOS flow cytometer (Beckman Coulter) using the manufacturer’s software. The following cell subsets within the CD45+ lymphocyte gate were characterized: T cells, CD3+; CD4+ T cells, CD3+/CD4+; activated T cells, CD3+/HLA-DR+; CD8+ T cells, CD3+/CD8+; naïve CD4+ T cells, CD3+/CD4+/CD45RA+; memory CD4+ T cells, CD3+/CD4+/CD45RO+; B cells, CD19+; NK cells, CD16+/CD56+; T-cell receptor α/β, TCR α/β; T-cell receptor *γ*/*δ*, TCR *γ*/*δ*; regulatory (Treg^low^) CD4+ T cells, CD3+/CD4+/CD25+/CD127+low; effector (Treg^high^) CD4+ T cells, CD3+/CD4+/CD25-/CD127+high. All antibodies were obtained from Beckmann Coulter (Krefeld, Germany), except for antibodies against TCR α/β and TCR *γ*/*δ*, which were from Miltenyi Biotec (Bergisch Gladbach, Germany). For the discrimination of live and dead cells, samples were incubated with 7-aminoactinomycin D (7-AAD, BD Biosciences, Heidelberg, Germany) directly prior to analysis.

### 2.5. Detection of Immunoglobulins and Soluble Interleukin 2 Receptor (sIL2R)

In order to quantify immunoglobulins of the classes IgA, IgE, IgG, and IgM and the level of soluble interleukin 2 receptor (sIL2R) in patients after HSCT, Immunoglobin Isotyping and IMMULITE^®^ 2000 IL2R System (Siemens Healthcare Diagnostics GmbH, Erlangen, Germany) was used according to the manufacturer’s instructions.

### 2.6. Preparation of Virus-Specific Donor T Cells for Adoptive Transfer

We selected partially HLA compatible third-party donors (8/10 and 6/10 HLA low resolution (single-field resolution); 6/10 and 6/10 high resolution (two-field resolution)) from the pre-examined T-cell donor registry alloCELL (www.allocell.org accessed on 20 February 2023). In addition to the HLA type of the patient, we considered the HLA type of the donor and excluded the mismatched HLA antigens to avoid patient-specific immunization. Donor pretesting was performed using IFN-γ Cytokine Secretion Assay (CSA) as described, with overlapping peptide pools covering LT and VP1 proteins of BKV as well as CMVpp65 and with EBV EBNA-1/EBV-Select (all Miltenyi Biotech, Bergisch Gladbach, Germany) [28,29]. Manufacturing of clinical-grade BKV-specific CD4+ and CD8+ T cells (VSTs) was performed on a CliniMACS Prodigy device using MACS GMP Peptivators LT and VP1 in combination and the IFN-γ Cytokine Capture System (Miltenyi Biotech, Bergisch Gladbach, Germany). For multivirus-specific T-cell products, the GMP peptide pools CMVpp65 and EBV EBNA-1/EBV-Select were additionally used (all Miltenyi Biotech). Sterile (aerobic and anaerobic) and quality controls such as the measurement of viability and determination of CD3+/IFN-γ+/− T-cell counts were performed on both the starting material and the final products, as described [28,29,30,31]. The patients received fresh and cryopreserved VSTs from a single manufacturing process each. The median viability of total CD3+ T cells prior to treatment was 90% (range 63–96%), as determined by 7-AAD staining. Every cycle of VSTs contained 25,000 CD3+ T cells/kg body weight, which were monospecific in two patients (against BKV), bi-specific in two patients (against BKV and CMV), and tri-specific in three patients (against BKV, CMV, and EBV).

### 2.7. Statistical Analysis

Data were analyzed using GraphPad Prism 8.4.2.679 (San Diego, CA, USA). ELISpot responses at the first time point (first dataset) and maximum responses of HSCT patients were compared by Wilcoxon matched pairs test. Spearman test was used to correlate ELISpot results with numerical variables and the Mann–Whitney test was used to analyze the impact of categorical variables. If not otherwise stated, median values are indicated. Two-sided *p*-values <0.05 were considered significant.

## 3. Results

### 3.1. ELISpot Responses in Patients with BKV-Related Cystitis and Control Patients

**Figure A2:**
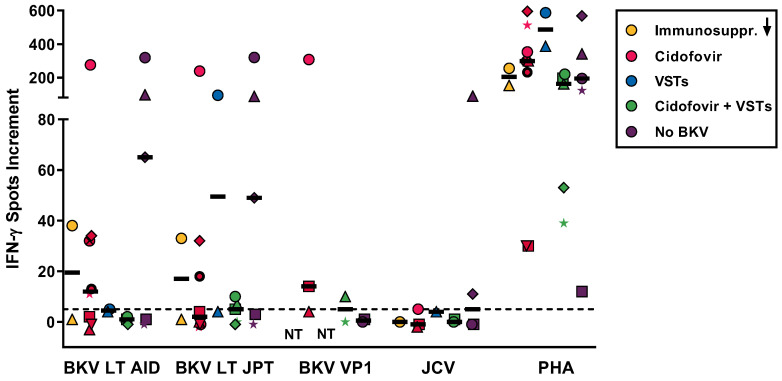
Cellular responses towards BK virus (BKV) large T (LT) peptides (from AID or JPT), as well as towards BKV viral protein 1 (VP1) and JC virus (JCV) peptides. Cell cultures stimulated with phytohemagglutinin (PHA) served as a positive control. The graph shows data on the first timepoint in hematopoietic stem cell transplant recipients with BKV infection, in whom only immunosuppression was reduced (*n* = 2), who was treated by cidofovir (*n* = 8), who received virus-specific T cells (VSTs) (*n* = 2), and who received cidofovir plus VSTs (*n* = 5). For comparison, we present the results of transplant recipients who suffered from cystitis that was not related to BKV (control group, *n* = 5). Each patient is depicted by an individual symbol. The values correspond to the first timepoint, as shown in Figure 1, which is a median of 28 days after the onset of cystitis. Data are indicated as spots increment, i.e., BKV-specific spots minus negative control. Solid horizontal lines indicate median values. The dotted horizontal line indicates the cut-off for positive responses (five spots increment). NT, not tested. BKV LT AID—BKV large T peptides (from AID), BKV LT JPT—BKV large T peptides (from JPT), BKV VP1—BKV viral protein 1 peptides.

Titration experiments and time kinetics could define 400,000 PBMCs per 1-day cell culture, 1.3 µg/mL per peptide of BKV LT (AID), or 1 µg/mL per peptide of BKV LT (JPT) as optimal for clinical application (Appendix A). BKV VP1 and JCV were used as recommended by the manufacturer, without titration. Applying these conditions, we compared 17 HSCT patients with BKV-related cystitis to five HSCT patients with cystitis that was not caused by BKV, who served as controls. In patients with and without BKV-related cystitis, the responses to the BKV LT peptides were overall at a similar level (Appendix B, Figure A2). However, in HSCT recipients with BKV-related cystitis, responses to BKV VP1 tended to be higher than in the controls without BKV. Vice versa, in recipients with BKV-related cystitis, responses to JCV tended to be lower than in patients without BKV-related cystitis (median of 0 vs. 5 spots increment). Two patients without BKV-related cystitis had 11 and 90 spots increment after stimulation with JCV peptides, indicating that cystitis could have been caused by another polyomavirus like JCV.

### 3.2. Assessment of Treatment Responses

We divided the 17 HSCT patients with BKV-related cystitis by treatment group (with reduction of immunosuppression only, with cidofovir only, with VSTs only, and with cidofovir and VSTs; Table 2) and assessed whether viral load decreased and/or T-cell responses were detectable. As BKV LT from JPT yielded overall the most robust T-cell response and was available throughout the whole study, the further evaluation of T-cell responses is related to this antigen. In the two patients with reduction of immunosuppression only, we detected a decrease in viral load in serum and/or urine and measured BKV-specific T cells. All eight patients treated with cidofovir only showed a reduction in the viral load. Half of them displayed specific T-cell responses. In the two patients treated with VSTs only, we observed a reduction in the viral load and could detect specific T-cell immunity. Five patients were treated with cidofovir and VSTs. Three of them showed a reduction in the viral load and four showed a BKV-specific T-cell response. Thus, six out of seven patients receiving VSTs had a detectable T-cell response, as did 6 out of 10 who did not receive VSTs.

### 3.3. Individual Time Courses of BKV-Specific ELISpot Responses and BK Viral Load

We present here individual courses of HSCT patients with BKV-related cystitis, including BKV-specific T-cell responses, BK viral load, antiviral treatment, immunosuppressive drugs, and time of transplantation and cystitis. The maximum follow-up was 1890 days (63 months) after the last reduction of immunosuppressive drugs, 1083 days (36 months) after treatment with cidofovir, 380 days (13 months) after VSTs, and 910 days (30 months) after VSTs plus cidofovir.

**Figure 1:**
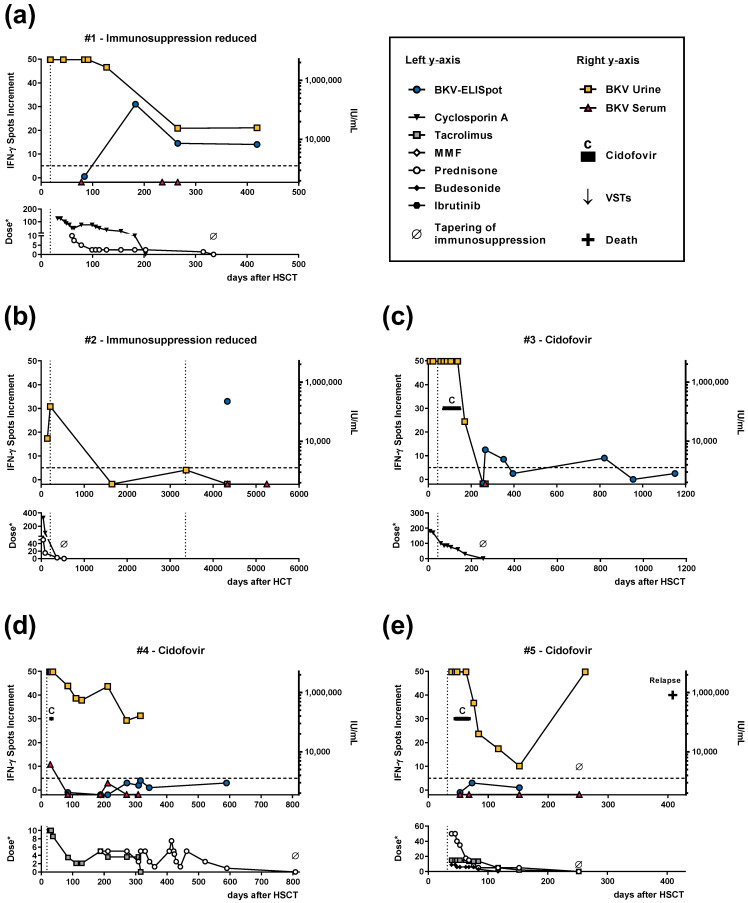

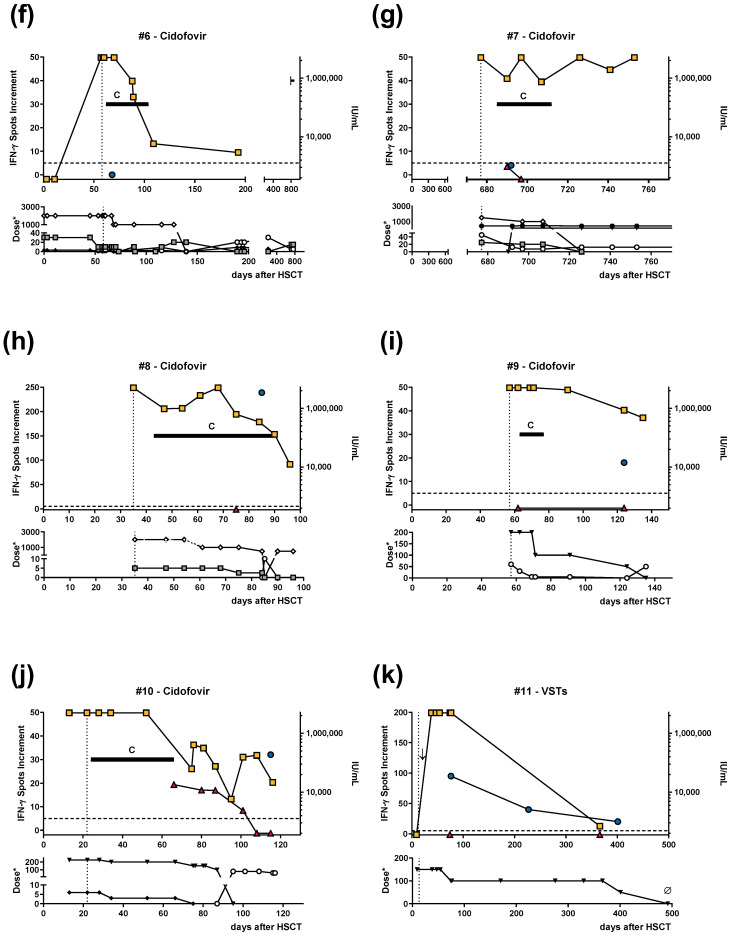

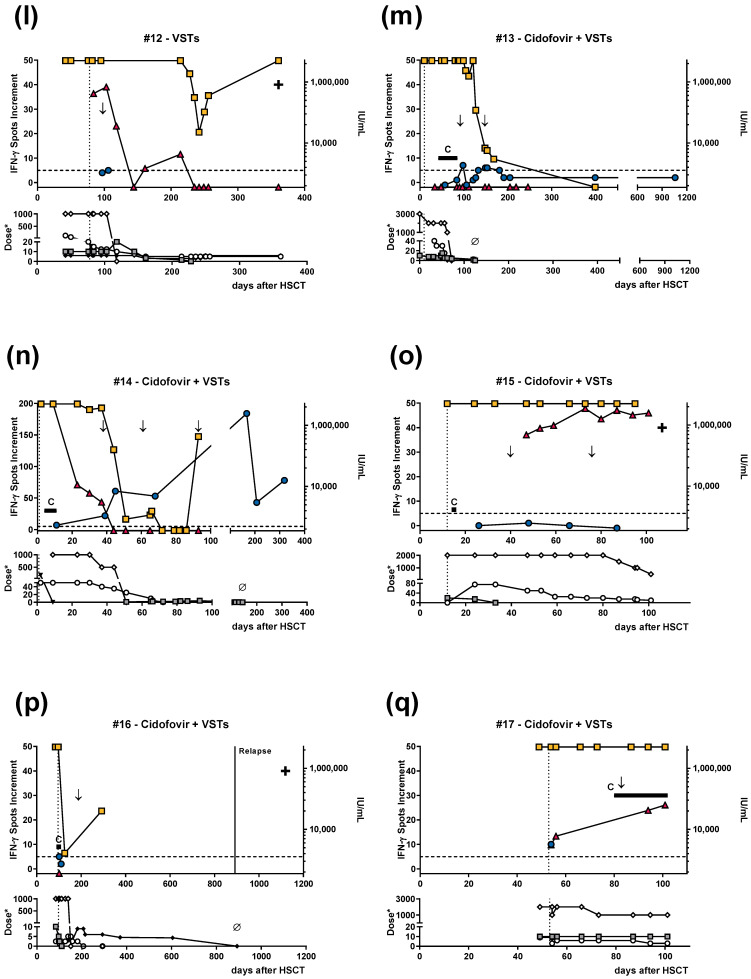
Follow-up data of hematopoietic stem cell transplant (HSCT) recipients who suffered from BK virus (BKV)-related cystitis. The dotted vertical line indicates the onset of BKV-related cystitis. The patients were either treated only with a reduction of their immunosuppressive drugs (**a**,**b**); with cidofovir (**c**–**j**); with BK virus-specific T cells (VSTs), as indicated by an arrow (↓) (**k**,**l**); or with a combination of cidofovir and VSTs (**m**–**q**). Cellular responses towards BKV large T (LT) peptides (from JPT) are indicated on the upper left *y*-axis as spots increment, i.e., BKV-specific spots minus negative control. The dotted horizontal line indicates the cut-off for positive responses (five spots increment). The upper right *y*-axis shows the viral load in urine and serum. With one exception, the lower left *y*-axis indicates the dose of immunosuppressive drugs as mg per day (*). To be visible, the dose of tacrolimus is given as mg * 10 per day. MMF, mycophenolate mofetil.

In two patients, only immunosuppression was reduced. In one of these HSCT patients (#1), in whom cystitis occurred at day 18 after transplantation (shown by a dotted vertical line), BKV-specific cellular immune responses increased to 31 spots increment after immunosuppression was reduced, at day 183 after HSCT (Figure 1a). Immunosuppressive treatment was tapered at day 355 after HSCT, when BKV-specific cellular responses could be detected. BK viral load in the urine decreased at the time when we observed the maximum cellular response. In the serum, BK viral load was always below the LLQ of 360 IU/mL. The second patient (#2) suffered from BKV-related cystitis on day 210 and 3359 (9.2 years) after HSCT (Figure 1b). After the first BKV infection, immunosuppression was reduced and, at the time of the second BKV infection, the patient did not receive any immunosuppressive therapy. On day 4332 after HSCT (i.e., 32 months after the last infection), we could detect strong BKV-specific cellular immunity (33 spots increment) and, at month 32 and 63 after the last infection, we detected the absence of BK viral load in urine and serum. Of note, as this patient suffered from late-onset cystitis, the cellular immune function cannot be compared to those patients with typical early-onset cystitis. The rather high spot number could thus also reflect the reconstitution of cellular immune function. As expected, his T-cell response was higher than the median of the total cohort.

Eight patients (#3–10) were treated with cidofovir and we detected BKV LT-specific cellular immunity (at least 5 spots increment) in four of these patients after treatment (#3 and #8–10) (Table 2, Figure 1c,h–j). Four of these eight patients received a graft from a haploidentical donor (#3, 6, 8, and 10). Of note, the patients with detectable BKV-specific immunity were the youngest patients and two of them were female. However, immunosuppression was usually reduced in parallel, and could thus also have augmented specific cellular immunity. In the first patient (#3), BKV-specific ELISpot responses (13 and 9 spots increment) were detectable on day 115 and day 670 (month 22) after treatment with cidofovir, i.e., on day 266 and 821 after HSCT (Figure 1c). The cellular responses were below the cut-off (maximum of three and four spots increment) in the other two patients (#4 and #5) (Figure 1d,e). BK viral load in the urine became undetectable (Figure 1c) or declined (Figure 1d,e) in the follow-up after treatment with cidofovir. BK viral load in the serum was either undetectable (Figure 1c,e) or could be observed only at a low level (Figure 1d). Unfortunately, one patient (#5) died as a result of relapse at day 408 after HSCT (Figure 1e). The remaining five patients (Figure 1f–j) were also tested for BKV-specific cellular immunity after having received cidofovir treatment for BKV-related cystitis. Whereas the patients tested at day 6 and 7 after the initiation of treatment, i.e., at day 68 and 692 after HSCT (#6 and #7, respectively), did not show BKV-specific cellular immunity (zero and four spots increment), those tested on days 42, 61, and 91 after the initiation of treatment (#8–10) displayed strong positive responses (239, 18, and 32 spots increment, respectively). The latter three patients were tested on days 85, 124, and 115 after HSCT.

One out of two patients who received VSTs but not cidofovir (#11) showed up to 95 spots increment to the ELISpot on day 55 after VSTs, which is on day 76 after HSCT (Figure 1k). In this patient, follow-up until day 401 after HSCT (day 380 after VSTs) indicated that BKV-specific cellular immunity declined, but was still rather strong (20 spots increment), although the patient received cyclosporin A. In the other patient receiving VSTs only (#12), cellular immunity was weakly positive on day 8 after receiving VSTs, i.e., on day 106 after HSCT (five spots increment) (Figure 1l). Whereas BK viral load decreased thereafter in the serum, the viral load in the urine remained high for more than 100 days. After a minimum on day 242 after HSCT, BK viral load in the urine increased again and the patient finally died on day 361 from septic shock due to *Pseudomonas* infection.

Five patients received cidofovir plus one to three doses of VSTs (#13–17). The first patient (#13) showed an increase in BKV-specific cellular immunity after he was treated with cidofovir and the first dose of VSTs, on day six after VSTs, i.e., day 98 after HSCT (Figure 1m). The maximum response was seven spots increment. After the second dose of VSTs, however, the increase in cellular immunity was only minor. Nevertheless, the BKV-specific response on day 910 after VSTs was still slightly higher that prior to VSTs (2 vs. −1 spot increment). In parallel, BK viral load in the urine decreased after VSTs. The second patient (#14) already showed BKV-specific cellular immunity at baseline, on day 35 after HSCT, and prior to VSTs (seven spots increment), which increased on day 35 after initiation of treatment with cidofovir (22 spots increment) and further increased after receiving the first and third dose of VSTs (61 and 184 spots increment, respectively) (Figure 1n). The data after the second dose, however, indicate no clear change. As compared with the baseline prior to VSTs, BKV-specific cellular responses were still increased on day 226 (month 7.5) after the third dose of VSTs, which was day 319 after HSCT (78 vs. 7 spots increment). Parallel to the increase in specific immunity, BK viral load in urine and serum declined. The third patient (#15) did not show BKV-specific immunity (maximum of one spot increment on day 48 after HSCT) (Figure 1o). Thus, despite treatment with cidofovir and two doses of VSTs, cellular immunity was absent and the BK viral load was very high. The patient finally died on day 107 after HSCT, also from septic shock due to *Pseudomonas* infection. The fourth patient (#16) showed only a weak effect of cidofovir treatment on BKV-specific cellular immunity (five and two spots increment on day 2 and 11 after cidofovir, respectively, i.e., on day 101 and 110 after HSCT) (Figure 1p). However, BK viral load in the urine was lower after treatment. On day 1119 after HSCT, the patient died from relapse. The fifth patient (#17, Figure 1q) suffered from BKV-related cystitis on day 53 after HSCT. He already showed BKV-specific cellular immunity prior to treatment with cidofovir and VSTs, on day 54 after HSCT (10 spots increment). BK viral load in the urine also exceeded the upper limit of detection of 5 million IU/mL on day 88 and 94 after HSCT, i.e., 8 and 14 days after initiation of treatment with cidofovir, respectively, and 5 and 11 days after VSTs, respectively. Unfortunately, we could not test BKV-specific cellular immunity after treatment.

Taken together, BKV-specific cellular immunity could be observed until month 32 after the last BKV-related cystitis in a patient with reduction of immunosuppressive drugs (patient #2), until month 22 after treatment with cidofovir (the last follow-up of patient #3 was month 36), until month 13 after VSTs (patient #11,) and until day 226 (month 7.5) after VSTs plus cidofovir (patient #14). In 12 out of 17 HSCT patients, BKV LT-specific cellular immunity was detectable before and/or after treatment for BKV-related cystitis. Two further patients showed increasing cellular responses after treatment with cidofovir (maximum of three and four spots increment). In the majority of cases, an increase in specific cellular immunity was observed parallel to a decrease in BK viral load. The patients who finally died (#5, #12, #15, and #16) all showed rather weak cellular responses to BKV LT (one to five spots increment). Two patients with poor responses to BKV LT (#7 and #15) showed a positive response to BKV VP1 peptides, either after treatment with cidofovir or with cidofovir and VSTs (14 and 210 spots increment, respectively). Patient #7 suffered from severe acute/chronic GvHD grade 3, respectively, and severe hemorrhagic BKV-associated cystitis. Under combined systemic immunosuppression with ibrutinib and biweekly extracorporeal photopheresis therapy, GvHD stabilized or showed an improvement, with concomitant regression of cystitis and an improvement in immune reconstitution. The patient with acute GvHD grade 3 who received PT/Cy (#15) developed severe BKV-associated cystitis with acute renal failure, having previously suffered from chronic kidney disease. Switching immunosuppression to a tacrolimus-free regimen and intravesical cidofovir therapy and VSTs resulted in symptom relief, but persistent dysuria.

### 3.4. Time Course of CMV- and EBV-Specific ELISpot Responses

**Figure 2:**
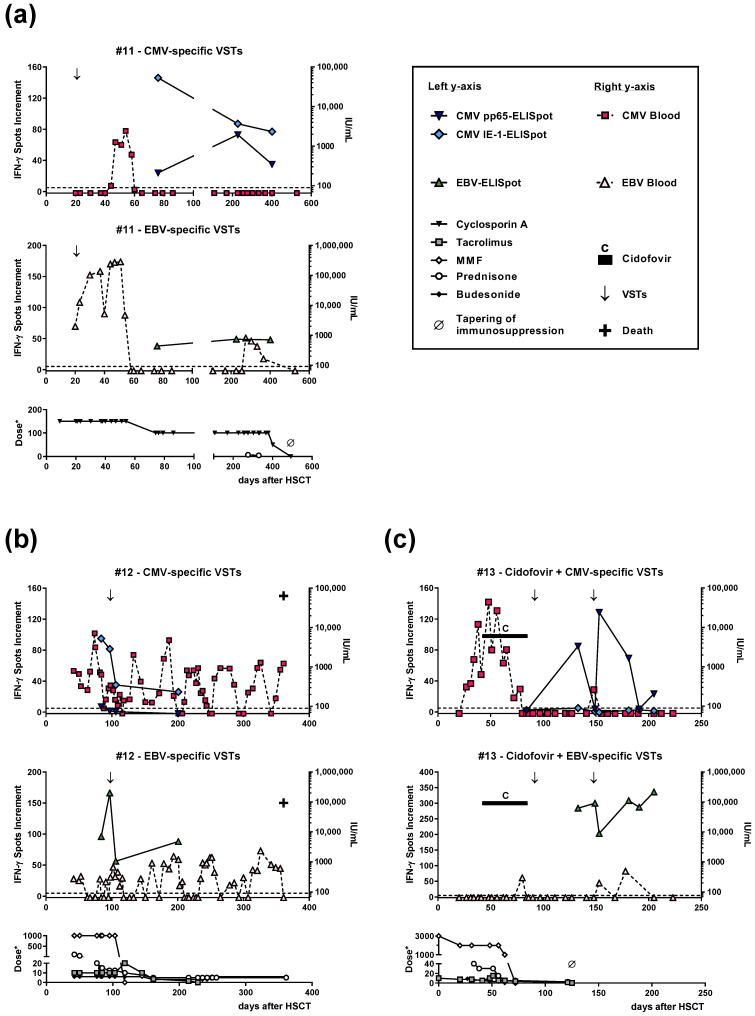
Follow-up data of hematopoietic stem cell transplant (HSCT) recipients who received tri-specific virus-specific T cells (VSTs), directed against BK virus, cytomegalovirus (CMV), and Epstein–Barr virus (EBV). The graphs show cellular in vitro responses against CMV phosphoprotein 65 (pp65) and immediate early-1 (IE-1) and EBV and viral load of CMV and EBV. Panel (**a**) corresponds to the patient shown in Figure 1k, panel (**b**) corresponds to Figure 1l, and panel (**c**) corresponds to Figure 1m, where immunity against BKV is displayed. Infusion of VSTs is indicated by an arrow (↓). The dotted horizontal line indicates the cut-off for positive responses (five spots increment). Cellular responses are shown on the upper left *y*-axis as spots increment, i.e., CMV- or EBV-specific spots minus negative control. The upper right *y*-axis shows the viral load in whole blood. With one exception, the lower left *y*-axis indicates the dose of immunosuppressive drugs as mg per day (*). To be visible, the dose of tacrolimus is given as mg * 10 per day. MMF, mycophenolate mofetil.

Three patients (#11–13) received tri-specific VSTs and were followed up not only for their BKV-specific immunity, but also for their CMV- and EBV-specific immunity (Figure 2). In patient #11 (Figure 2a), the CMV viral load in whole blood intermittently increased and was undetectable since day 44 after VSTs. EBV viral load increased after VSTs and declined approximately three weeks thereafter. In the long term, we observed strong T-cell responses towards CMV pp65, CMV IE-1, and EBV lysate. In patient #12 (Figure 2b), CMV and EBV viral load in the blood showed median values below the lower limit of quantification (65 IU/mL for CMV and 150 IU/mL for EBV). However, both viruses were detectable intermittently throughout the whole follow-up period. Cellular responses to CMV pp65 and CMV IE-1 and to EBV were clearly detectable prior to and post VSTs. In patient #13 (Figure 2c), the CMV viral load in the blood became undetectable after VSTs’ infusion and EBV was (nearly) undetectable throughout the study. After the infusion of two cycles of VSTs, we observed an increase in CMV-specific cellular immunity. Moreover, we observed strong cellular immunity towards EBV after both cycles. Thus, the three patients who received tri-specific VSTs all showed cellular responses against CMV and EBV. Two further patients received bi-specific VSTs (against BKV and CMV), but were unfortunately not followed up for CMV-specific cellular immunity.

Moreover, we compared the strength of BKV-specific cellular responses in three patients who received tri-specific VSTs (against BKV, CMV, and EBV, patients #11–13), in one patient who received bi-specific VSTs (against BKV and CMV, patient #16), and in two patients who received monospecific VSTs (only against BKV, patients #14–15). We here considered the maximum response as displayed in Table 2, which was 95, 5, and 7 spots increment after tri-specific VSTs; five after bi-specific VSTs; and 184 and 1 after monospecific VSTs.

### 3.5. Correlation of BK-Virus-Specific Cellular Immunity with Co-Variates

**Figure A3:**
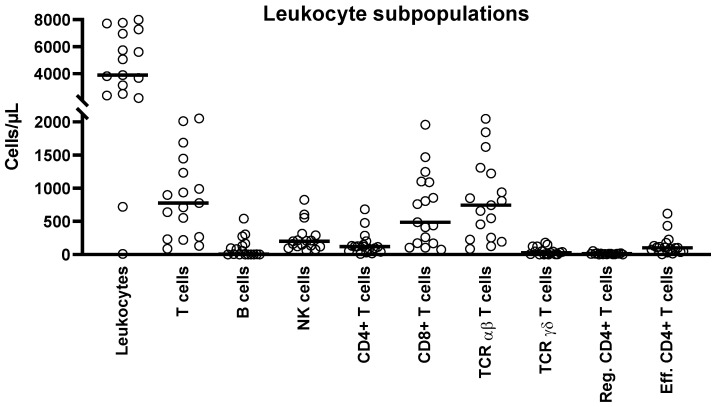
Distribution of leukocyte subpopulations in the patient cohort shown in Table A1. This figure contains the first dataset of each HSCT recipient with BKV-related cystitis. The median interval between HSCT and study inclusion (first dataset) was 85 days (range 26 days–12 years). Median values are indicated by horizontal lines. Reg.—regulatory, Eff.—effector.

**Figure A4:**
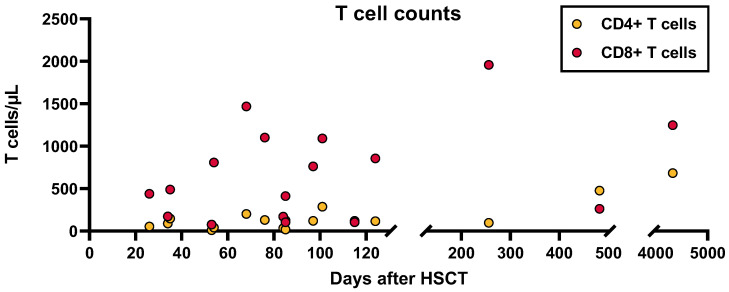
Correlation of the interval between HSCT and study inclusion (days after HSCT) and CD4+ and CD8+ T-cell counts in the patient cohort shown in Table A1. Spearman analysis indicated that CD4+ T-cell counts showed a stronger correlation with days after HSCT (*r* = 0.46, *p* = 0.06) than CD8+ T-cell counts (*r* = 0.28, *p* = 0.3).

Using the first dataset in each patient with BKV-related cystitis (first timepoint as indicated in Table 1), we analyzed whether patient covariates correlated with ELISpot responses towards BKV LT peptide (JPT), using 400,000 PBMC in a 1-day cell culture (Appendix B, Table A1). As expected, leukocyte numbers showed a wide range (Appendix B, Figure A3) and cell counts were dependent on the time to HSCT (Appendix B, Figure A4). Spearman analysis yielded a positive correlation with the absolute number of CD4+ T cells (*r* = 0.52, *p* = 0.03) and with the absolute number of effector CD4+ T cells (*r* = 0.51, *p* = 0.04). Moreover, the glomerular filtration rate correlated positively with ELISpot responses (*r* = 0.64, *p* = 0.01) and serum creatinine and urea correlated negatively (*r* = −0.51, *p* = 0.04 and *r* = −0.52, *p* = 0.03, respectively). However, the time to HSCT only by trend correlated with BKV-specific ELISpot responses (*r* = 0.28, *p* = 0.3).

### 3.6. Comparison of BKV-Specific ELISpot Responses in Hematopoietic Stem Cell Transplant Recipients with BKV-Related Cystitis and Healthy Controls

**Figure 3:**
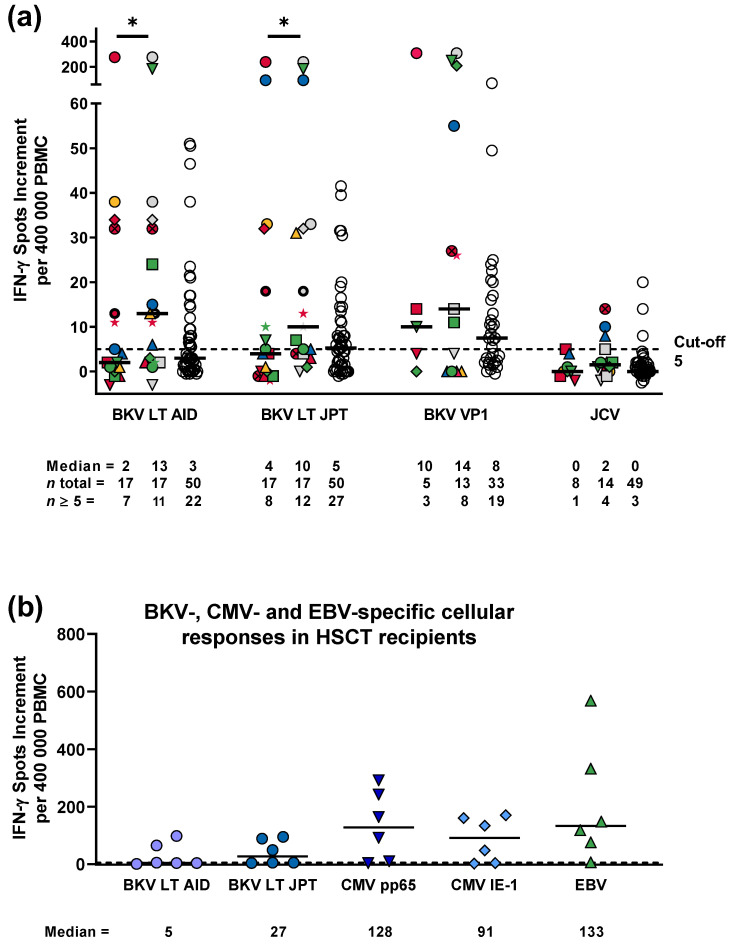
Specific cellular immunity against peptides of the BK virus (BKV). (**a**) Responses against large T (LT) and viral protein (VP) 1 peptides of BKV and against a structurally related polyomavirus, the JC virus (JCV), in 17 hematopoietic stem cell transplant (HSCT) recipients with BK virus-related cystitis (coloured symbols) and in 50 healthy controls (HC, white circles). The first dataset in each patient is shown as the left panel, with the maximum response as the middle panel. Each patient is depicted by an individual symbol. Treatment groups are colour-coded (yellow: with reduction of immunosuppression only, red: with cidofovir only, blue: with VSTs only, and green: with cidofovir and VSTs). For patients tested only once, the first set of data is equal to the maximum response, which is shown as a grey symbol (and which is not considered for comparison of the first and maximum response). Please note that only part of the datasets contained VP1 and JCV. One-day cell cultures were performed with 400,000 peripheral blood mononuclear cells and the antigen concentrations defined as optimal. (**b**) Specific cellular immunity against LT peptides of BKV, cytomegalovirus (CMV) phosphoprotein 65 (pp65), CMV immediate early-1 (IE-1), and Epstein–Barr virus (EBV), analyzed in parallel in six HSCT recipients with BKV-related cystitis. Each viral antigen is indicated by a specific symbol and color. Median values are indicated with solid horizontal lines. The dotted horizontal line indicates the cut-off for positive responses (five spots increment). * *p* < 0.05 (Wilcoxon matched pairs test). BKV LT AID—BKV large T peptides (from AID), BKV LT JPT—BKV large T peptides (from JPT), BKV VP1—BKV viral protein 1 peptides.

Using BKV LT and VP1 peptides as well as JCV peptides as stimuli, median responses in HSCT patients with BKV-related cystitis and healthy controls were similar, when considering the first dataset each (Figure 3a). In 8 out of 17 patients with BKV-related cystitis (47%) and in 27 out of 50 controls (54%), cellular responses to BKV LT (JPT) were detectable. The respective numbers for BKV VP1 were 3 out of 5 (60%) and 19 out of 33 (58%). Considering the maximum response each, usually after treatment for BKV cystitis, these numbers increased in the HSCT recipients to 12 out of 17 (71%) for BKV LT (JPT) (*p* < 0.05, compared with the first dataset) and to 8 out of 13 (62%) for BKV VP1. The median spots increment in the first dataset were 4 for BKV LT (JPT) and 10 for VP1, at maximum the respective numbers were 10 and 14.

### 3.7. Correlation of ELISpot Responses to Various BK Virus and JC Virus Peptide Pools

Spearman analysis indicated that, in 17 HSCT recipients with BKV-related cystitis and in 50 healthy controls, responses to the BKV LT and VP1 peptide pools showed a highly significant correlation (*r* > 0.7, *p* < 0.0001) (Appendix B, Table A2). However, in both cohorts, we could not observe correlation between responses to BKV and JCV (*r* = 0.12–0.25 and *p* = 0.2–0.6, depending on the BKV peptide mix), arguing against major cross-reactivity. Similarly, the responses to BKV and to the positive control showed no correlation in the HSCT recipients (*r* = −0.15 to −0.08), but weak positive correlation in the healthy controls (*r* = 0.15 to 0.40, *p* = 0.03 to 0.03). Thus, in HSCT patients, higher cellular responses against BKV did not correlate with a generally stronger T-cell function (response against the T-cell mitogen PHA).

### 3.8. Comparison of ELISpot Responses to BK Virus and Herpes Viruses

To further assess the strength of cellular responses, we stimulated PBMCs from six HSCT recipients in parallel with BKV, CMV pp65, CMV IE-1, and EBV antigens. To allow a direct comparison, we determined spot numbers per 400,000 PBMCs. As compared with responses to BKV LT (JPT), responses to CMV pp65, CMV IE-1, and EBV were approximately five-, three-, and fivefold higher, respectively (Figure 3b).

## 4. Discussion

The current study indicates that more than two-thirds of the HSCT recipients treated for BKV-related cystitis displayed detectable BKV-specific cellular responses, as determined by IFN-γ ELISpot. In recipients treated with VSTs, 6 out of 7 displayed specific T-cell responses to BKV LT and that number in those without VSTs was 6 out of 10. As shown in patient #14, BKV-specific cellular immunity could be detected as early as day 35 after HSCT (before VSTs) and remained elevated (78 vs. 7 spots increment) until day 226 after VSTs, indicating the high sensitivity of the ELISpot assay in the HSCT setting too.

Two previous studies on HSCT recipients treated with BKV-specific VSTs reported monitoring of specific cellular immunity [17,19]. Tzannou et al. [17] performed BKV-specific ELISpot assays until week 12 after infusion of BKV-specific VSTs and observed that 7 out of 16 HSCT patients (44%) showed an increase in specific T-cell responses. If we change our cut-off for positive reactions and take 10 spots increment instead of 5, four patients with weak positive responses would have been classified as negative, which results in a positivity rate of three out of seven, similar to the previous data [17]. However, two of these four patients (#12 and # 13) showed a decrease in viral load after VSTs, indicating BKV-specific T-cell immunity. In four of the patients from the previous study [17], follow-up data until week 3, 4, 6, and 12 after VSTs were presented, respectively, indicating an overall declining frequency of VSTs. After stimulation with epitope-specific peptides, Tzannou et al. [17] detected BKV-specific T cells derived from the VSTs in three patients at the latest time point of follow-up, at week 3, 4, and 12. However, in the fourth patient, VSTs were no longer detectable at week 6. Moreover, Olsen et al. [19] measured the frequency of BKV-reactive T cells by intracellular cytokine assays in 32 patients and presented data until month 3 after VSTs. They showed a peak in spot numbers at day 28. Using flow chimerism assays in three patients, they could further detect T-cell responses derived from the infused BKV-VSTs for up to three months. Extending these previous data, the current study presents a longer follow-up in a subset of patients, where BKV-specific cellular immunity was monitored up to day 910 after VSTs (patient #13). In this patient, at least five spots increment were found until day 30 after VSTs. At day 43, however, numbers declined to two spots increment. This number remained constant until day 910 and was higher than the baseline, prior to VSTs (−1 spot increment). In patient #11, we could still detect BKV-specific T cells on day 380 (week 54) after VSTs (20 spots increment). Moreover, in patient #14, we detected BKV-specific T cells at day 226 (week 32) after VSTs (78 spots increment), when spot numbers were still 11-fold higher than at baseline prior to VSTs. Thus, our data show that, after infusion of VSTs, the frequency of BKV-specific T cells increased for more than eight months (patient #14), possibly even for more than 2.5 years (patient #13). However, unlike the previous studies [17,19], we did not determine if cellular BKV immunity was derived from the VSTs. Nevertheless, long-term BKV-specific T-cell immunity—either adoptively transferred or by the patient’s own immune system—should control viral replication.

The BKV-related cystitis often occurs between 2 and 12 weeks (up to months) after HSCT and typically starts in the peri-engraftment period, when cystitis is caused by the toxicity of the conditioning regimen, e.g., with cyclophosphamide or total body irradiation [14]. Other viruses such as CMV, herpes simplex virus, adenovirus, and JCV, as well as bacterial infections and non-infectious etiologies (especially hemorrhage, catheter injury, and so on), should be considered as additional causes. Moreover, subclinical urotoxic exposure may damage the urothelial cell layer, causing local inflammation, favoring BKV replication owing to impaired antiviral immune control by cytotoxic T cells [32]. In particular, unrelated donor and haploidentical transplants, as well as transplant-associated complications such as GvHD, contribute to BKV pathogenesis, because of altered allogeneic immunity [33]. At many centers, the current management of BKV-related cystitis includes testing for viral reactivation only in symptomatic cases and in the presence of risk factors of BKV-related cystitis. Owing to the limited prophylactic and therapeutic options and the lack of an effective, clinically validated antiviral drug for the treatment of BKV-associated cystitis, a reliable diagnosis of specific antiviral immunity is very relevant. Of note, the two patients who were only treated with a reduction of immunosuppression both showed specific cellular responses thereafter. Although BKV viremia can predict cystitis, the positive predictive value of viremia remains low or uncertain. Recently, Laskin and coworker showed that screening for BKV viremia in children and young adults after HSCT identifies asymptomatic recipients at risk for kidney disease and reduced survival. Their data suggest potential changes to clinical practice, including prospective monitoring for BKV viremia, and to test for virus-specific T cells, in order to identify early BKV replications [15].

ELISpot was suitable to detect BKV-specific immunity already on day 35 after HSCT (patient #14), when T-cell immunity is usually considered as severely impaired. In the HSCT patients, median T-cell responses towards BKV were similar to responses in healthy controls, considering the first dataset. Of note, cellular responses towards BKV LT and VP1 peptides in 50 healthy controls in the current study were in a similar range to previous reports including 10 healthy individuals [34] (5 and 8 spots increment vs. 10 and 10 spots increment per 400,000 PBMCs, respectively). After treatment for BKV-related cystitis, the rate of positive cellular responses increased in the HSCT recipients, most likely because a decreased dose of immunosuppressive drugs enhanced specific T-cell responses and infused VSTs persisted. Interestingly, in HSCT patients, the strength of T-cell responses against BKV did not correlate with a generally stronger T-cell response. The cellular response to BKV LT peptides (JPT) and to the T cell mitogen PHA even showed a negative correlation coefficient (*r* = −0.08). It may be assumed that BKV persistence is favored by generally weaker T-cell function and that BKV-related cystitis could have induced BKV-specific T-cell responses. In accordance with this assumption, we previously observed a similar phenomenon for CMV-specific cellular immunity [35]. The percentage of positive CMV-specific cellular responses was higher in HSCT recipients as compared with healthy controls. However, there is a second factor that influences cellular immune function in the opposite direction—the reconstitution of T-cell numbers and function after HSCT, which includes BKV-specific cellular immunity. Stervbo and coworker demonstrated in kidney transplanted patients an association between the resolution of BKV reactivation and reconstitution of BKV-specific CD4+ T cells. They followed T-cell receptor (TCR) single clone levels with multi-parameter flow cytometry and next-generation sequencing (NGS)-based CDR3 beta chain receptor repertoire analysis, and showed that the TCR repertoire diversity and exhaustion status of BKV-specific T cells affected the duration of viral clearance [36]. In line with these data, we found that the number of CD4+ T cells and effector CD4+ T cells significantly correlated with the magnitude of BKV-specific cellular responses, indicating their direct involvement in the antiviral cellular responses that we measured by ELISpot. Of note, CD4+ T cells are a major source of IFN-γ production and, as we used 15 and 20 mer peptides, it is most likely that CD4+ T cells were detected by our ELISpot assay. Supporting this assumption, Wilhelm et al. [37] showed an expansion of IFN-γ CD4+ T cells after stimulation with 15 and 27 mer BKV peptides. However, as the ELISpot assay used PBMCs and not T-cell subpopulations, we cannot prove this hypothesis. As expected, T-cell immunity in patients with better kidney function was stronger, as has been observed previously in kidney transplant recipients after vaccination [38]. Adoptively transferred VSTs generated from eligible donors could provide broad antiviral protection to recipients of HSCT, including infections from BKV or other virus-related pathogens with low side effects, and appears to be an effective approach to treat severe viral infection [17].

Of note, although BKV-specific immunity was clearly detectable in a subset of HSCT recipients, it was approximately three- to fivefold lower than CMV- or EBV-specific cellular immunity. In parallel to an increase in BKV-specific immunity, the BK viral load usually declined, which indicates that the T cells should be functionally active. In two out of three patients, a similar phenomenon was observed for CMV- and EBV-specific T-cell immunity and viral load (Figure 2a,c).

According to previous data, BKV viruria is detected in approximately 40% of patients after allogeneic HSCT [14,15,39]. In our current cohort from Essen, however, we observed BKV-related cystitis in less than 10% of the patients. Cyclosporin A, which may cause or aggravate cystitis, is rarely used as an immunosuppressive treatment at our center. It is possible that additional patients suffered from BKV infection, but not from BKV-related cystitis. Supporting this consideration, 6 out of 17 patients with BKV-related cystitis were treated with cyclosporin A.

The rather low patient number and the variation in the time points of measurement are a clear limitation of this study. Nevertheless, we believe that the data are of interest as we present a long-term follow-up and comprehensively describe not only ELISpot data, but also viral load, antiviral treatment, immunosuppressive drugs, and timing of transplantation and cystitis.

The current study indicates that a peptide pool of the BKV LT protein is a suitable stimulus for cellular in vitro assays in patients after HSCT. However, it may be reasonable to use various BKV antigens (LT and VP1) when assessing cellular immunity. In our cohort, a combined evaluation would have resulted in an increased frequency of positive BKV-specific cellular responses. VP1 appears as immunodominant in a subset of HSCT patients with BKV-related hemorrhagic cystitis.

## 5. Conclusions

Using an IFN-γ ELISpot assay, we detected BKV-specific cellular immunity in six out of seven HSCT patients with BKV-related cystitis treated with specific VSTs. ELISpot was highly sensitive. It was able to detect BKV-specific cellular immunity even early after transplantation and to measure increased specific immunity for more than seven months after VSTs. We thus propose ELISpot as a response marker in this patient cohort, in which we observed the median onset of BKV-related cystitis on day 35 after HSCT. BKV-specific cellular responses correlated with the absolute number of CD4+ T cells and effector CD4+ T cells, renal function, and general clinical condition.

## Figures and Tables

**Table 1:** Characteristics of 22 hematopoietic stem cell transplant recipients with cystitis.

ID	Sex	Age	MAC	ATG	Onset Cystitis	Days after Cystitis	BKV Cystitis	i.v. CIDO	Intravesical CIDO	VSTs
1	M	72	N	Y	18	66	Y	N	N	N
2	F	56	Y	Y	3359	973	Y	N	N	N
3	M	49	N	Y	44	212	Y	80 (2)	N	N
4	M	54	N	Y	18	67	Y	320 (1)	N	N
5	M	41	N	N	32	21	Y	110 (2)	N	N
6	M	70	Y	N	58	10	Y	240 (4)	N	N
7	F	24	N	N	677	15	Y	180 (4)	N	N
8	M	39	N	N	35	50	Y	230 (4)	N	N
9	F	27	Y	Y	57	67	Y	150 (3)	(1)	N
10	M	29	Y	N	22	93	Y	230 (5)	(4)	N
11	M	21	N	Y	13	63	Y	N	N	BCE (1)
12	M	37	N	N	78	19	Y	N	N	BCE (1)
13	M	50	N	N	10	47	Y	400 (3)	N	BCE (1)
14	M	77	N	Y	1	34	Y	240 (2)	N	B (3)
15	M	65	N	N	12	14	Y	N	(1)	B (2)
16	M	62	Y	Y	96	5	Y	360 (1)	N	BC (1)
17	M	74	Y	Y	53	1	Y	160 (4)	(1)	BC (1)
18	M	57	N	Y	929	7	N	N	N	N
19	M	50	Y	Y	65	1	N	N	N	N
20	M	48	N	N	628	0	N	N	N	N
21	M	63	Y	Y	27	57	N	N	N	N
22	F	71	Y	N	50	7	N	N	N	N

Age—patient age at the time of the first ELISpot (inclusion into the study), MAC—myeloablative regimen, ATG—anti-thymocyte globulin, onset cystitis—start of (last) cystitis (days after HSCT), days after cystitis—first timepoint of ELISpot analysis (days after onset of cystitis), M—male, F—female, Y—yes, N—no. The dose of cidofovir (CIDO) that was applied intravenously (i.v.) for the first cycle of treatment is given in mg (cycles). In one patient, the dose for the subsequent cycles was reduced because of impaired renal function (#13). Intravesical treatment contained 375 mg cidofovir and the number of cycles is indicated in brackets. Virus-specific T cells (VSTs) were applied at a dose of 25,000 CD3+ T cells per cycle (1–3), either directed against BKV only (B), against BKV and CMV (BC), or against BKV, CMV, and EBV (BCE).

**Table 2:** Assessment of treatment responses in 17 hematopoietic stem cell transplant recipients with BKV-related cystitis.

ID	Immuno- suppression ↓	CIDO	VSTs	CIDO + VSTs	VL ↓	T-Cell Response	Spots Increment	Day after HSCT
1	x				✓	+	31	183
2	x				✓	+	33	4332
3		x			✓	+	13	266
4		x			✓	∅	4	316
5		x			✓	∅	3	73
6		x			✓	∅	0	68
7		x			✓	∅	4	692
8		x			✓	+	239	85
9		x			✓	+	18	124
10		x			✓	+	32	115
11			x		✓	+	95	76
12			x		✓	+	5	106
13				x	✓	+	7	98
14				x	✓	+	184	167
15				x	∅	∅	1	48
16				x	✓	+	5	101
17				x	∅	+ *	10 *	54

CIDO–cidofovir, VSTs—virus-specific T cells, VL—viral load, spots increment—BKV-specific spots minus negative control, maximum of virus-specific IFN-γ spots after stimulation with BK virus (BKV) large T (LT) peptides (from JPT). The last column (day after HSCT—hematopoietic stem cell transplantation) indicates the timepoint at which T-cell responses were measured. With the exception of recipient #17, this T-cell response was measured after treatment. In recipient #17, VL remained above the upper limit of detection of 5 million IU/mL at day 5 and 11 after VSTs. * Spots prior to treatment. As spot numbers usually increased after treatment, it is assumed that the response after treatment should also be positive. Further information on patient treatment is given in Table 1 (ID #1–17) and more details on individual treatment responses are depicted in Figure 1. ↓—Reduction of immunosuppression or VL, x—This treatment was applied, ✓—BK viral load decreased, ∅—BK viral load did not decrease or BKV-specific T cells were undetectable after treatment, +—BKV-specific T cells were detectable after treatment.

**Figure A1:**
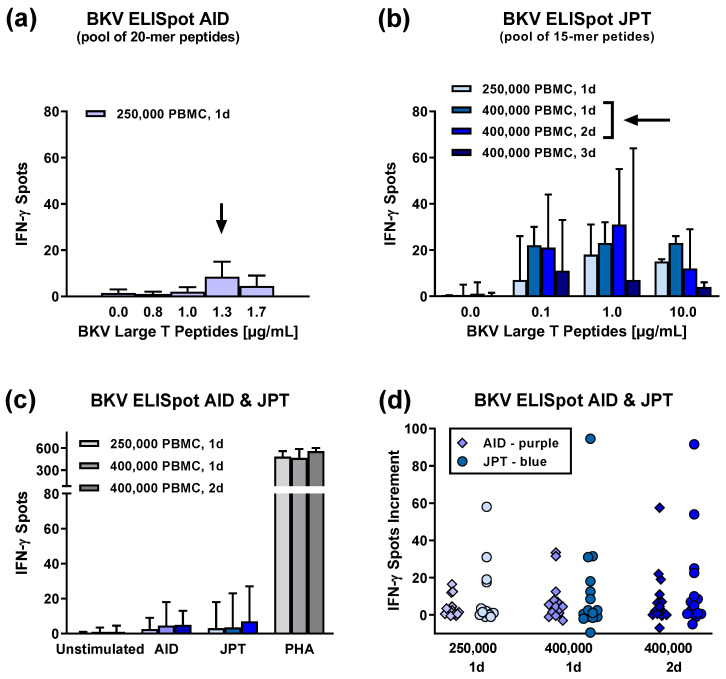
Optimization of ELISpot conditions to determine specific cellular immunity against BK virus (BKV) in patients after hematopoietic stem cell transplantation. Panel (**a**) shows the titration of 20 mer peptides of BKV large T (AID), using 250,000 peripheral blood mononuclear cells (PBMCs) in 1-day cell cultures (*n* = 2); panel (**b**) shows the titration of a 15 mer peptide of BKV large T (JPT) with 250,000 to 400,000 PBMCs in 1–3-day cell cultures (*n* = 3); and panel (**c**) shows data from 250,000 and 400,000 PBMCs and various culture durations with both BKV peptides used at optimal concentrations (1.3 and 1 µg/mL per peptide, respectively, indicated by an arrow) (*n* = 15). Each left column indicates data on 250,000 PBMCs and 1-day cell cultures, each middle column on 400,000 PBMCs and 1-day cell cultures, and each right column on 400,000 PBMCs and 2-day cell cultures. The graphs (**a**–**c**) show the median and 75% percentile. Panel (**d**) is based on the data as displayed in panel (**c**). It indicates individual values, given as spots increment, i.e., response towards BK virus (BKV) large T peptides (from AID or JPT) minus unstimulated controls (*n* = 15). PHA, phytohemagglutinin (positive control). In panel (**c**) and (**d**) the brightest color (either grey, blue or purple) indicates 250,000 PBMC and 1d culture, the intermediate color 400,000 PBMC and 1d culture and the darkest color 400,000 PBMC and 2d culture.

## Data Availability

The data presented in this study are available upon request from the corresponding author. The data are not publicly available owing to privacy restrictions.

## Appendix A

Initial titration experiments were performed with 250,000 PBMCs from HSCT recipients at 0.8–1.7 µg/mL per peptide of BKV large T (LT) from AID in 1-day cell cultures and showed a maximum response at a concentration of 1.3 µg/mL; this is the manufacturer’s recommended concentration (Figure A1a). Titration of 250,000 and 400,000 PBMCs with 0.1–10 µg/mL per peptide of BKV LT (JPT) in 1–3-day cell cultures indicated the strongest response for 400,000 PBMCs, a concentration of 1 µg/mL and 1–2-day cell cultures (Figure A1b). Again, the titration experiments were able to reconfirm the manufacturer’s recommended peptide concentration. Using the optimal peptide concentrations, we found that 1-day and 2-day cell cultures with 400,000 PBMCs gave similar results (Figure A1c,d), and we decided to use 1-day cultures for further experiments, which is more convenient for clinical use.

## Appendix B

**Table A1:** Spearman correlation analysis of ELISpot responses towards BKV large T peptides (JPT) in 17 hematopoietic stem cell transplant recipients with BKV-related cystitis.

Parameter	*r*	*p*
Age	−0.25	0.3
Interval to HSCT	0.28	0.3
Hemoglobin	−0.11	0.7
IgA	−0.06	0.8
IgE	−0.22	0.4
IgG	−0.25	0.3
IgM	0.06	0.8
sIL-2R	−0.19	0.5
Leukocytes	−0.22	0.4
T cells	0.22	0.4
B cells	0.25	0.8
NK cells	−0.06	0.8
CD4+ T cells	0.52	0.03
CD8+ T cells	0.19	0.5
TCRaβ T cells	0.24	0.4
TCRγδ T cells	0.26	0.3
Regulatory CD4+ T cells	0.40	0.1
Effector CD4+ T cells	0.51	0.04
GFR	0.64	0.01
Creatinine	−0.51	0.04
Urea	−0.52	0.03

This analysis considered the first dataset of each patient. Age—patient age at the time of the first ELISpot (inclusion into study), interval to HSCT—interval between hematopoietic stem cell transplantation and first ELISpot, sIL-2R—soluble interleukin 2 receptor, GFR—glomerular filtration rate.

**Table A2:** Spearman correlation analysis of ELISpot responses towards peptide pools of large T (LT) from AID and JPT, of BKV viral protein (VP) 1, and of JC virus (JCV).

		HSCT ^a^		HC ^b^	
Antigen 1	Antigen 2	*r*	*p*	*r*	*p*
BKV LT1 AID	BKV LT1 JPT	0.76	<0.0001	0.85	<0.0001
BKV LT1 AID	BKV VP1	0.74	<0.0001	0.73	<0.0001
BKV LT1 JPT	BKV VP1	0.75	<0.0001	0.84	<0.0001
BKV LT1 AID	JCV	0.12	0.5	0.12	0.4
BKV LT1 JPT	JCV	0.15	0.4	0.13	0.4
BKV VP1	JCV	0.13	0.6	0.25	0.2
BKV LT1 AID	PHA	−0.11	0.4	0.15	0.3
BKV LT1 JPT	PHA	−0.08	0.5	0.25	0.08
BKV VP1	PHA	−0.15	0.5	0.40	0.03
JCV	PHA	0.25	0.14	−0.10	0.5

^a^ Hematopoietic stem cell transplant recipients with BKV-related cystitis (*n* = 17 patients with 61 samples), ^b^ healthy controls (*n* = 50), PHA—phytohemagglutinin (positive control).
